# Elastic MCF Rubber with Photovoltaics and Sensing for Use as Artificial or Hybrid Skin (H-Skin): 1st Report on Dry-Type Solar Cell Rubber with Piezoelectricity for Compressive Sensing

**DOI:** 10.3390/s18061841

**Published:** 2018-06-05

**Authors:** Kunio Shimada

**Affiliations:** Faculty of Symbiotic Systems Sciences, Fukushima University, 1 Kanayagawa, Fukushima 960-1296, Japan; shimadakun@sss.fukushima-u.ac.jp; Tel.: +81-24-548-5214

**Keywords:** hybrid skin (H-Skin), solar cell, sensing, piezoelectricity, photovoltaics, natural rubber, electrolytic polymerization, magnetic cluster, magnetic field, magnetic compound fluid (MCF), artificial skin, robot, humanoid

## Abstract

Ordinary solar cells are very difficult to bend, squash by compression, or extend by tensile strength. However, if they were to possess elastic, flexible, and extensible properties, in addition to piezo-electricity and resistivity, they could be put to effective use as artificial skin installed over human-like robots or humanoids. Further, it could serve as a husk that generates electric power from solar energy and perceives any force or temperature changes. Therefore, we propose a new type of artificial skin, called hybrid skin (H-Skin), for a humanoid robot having hybrid functions. In this study, a novel elastic solar cell is developed from natural rubber that is electrolytically polymerized with a configuration of magnetic clusters of metal particles incorporated into the rubber, by applying a magnetic field. The material thus produced is named magnetic compound fluid rubber (MCF rubber) that is elastic, flexible, and extensible. The present report deals with a dry-type MCF rubber solar cell that uses photosensitized dye molecules. First, the photovoltaic mechanism in the material is investigated. Next, the changes in the photovoltaic properties of its molecules due to irradiation by visible light are measured under compression. The effect of the compression on its piezoelectric properties is investigated.

## 1. Introduction

Ordinary solar cells are very difficult to bend, squash by compression, or extend by tensile strength. This is because they are solid-state devices, generally made of silicon systems including nanocrystalline or non-crystalline chemical compounds, or organic polymer films, such as plastic polymers. Recently, studies on flexible solar power generators have attracted attention [1,2,3]; however, they have remained within the realm of solid-state materials, distinct from flexible materials, such as rubber. For example, they have been used to fabricate a thin solid film, even though they have no particular propensity for expansion or compaction in order to be characterized as bending materials; the amount of research on rubber-type solar cells remains limited [4,5].

The effect of rubber properties on simultaneously appearing photovoltaics and piezoelectricity are not yet reported. These effects have been investigated separately. If solar cell materials could be developed with elastic, flexible, and extensible properties, and could also exhibit piezoelectricity, they would be extremely effective for use as artificial skin installed over human-like robots or humanoids. The casing could generate electric power from solar energy and perceive all force or temperature changes. Other varied engineering applications would also become feasible. In addition, such hybrid properties of both photovoltaics and piezoelectricity, or piezoresistivity would not require a power supply or batteries.

This new type of artificial skin, having all the above properties, has been named “hybrid skin (H-Skin)”. Human skin, humanoid skin, or outer layer of robot skin made of artificial material integrated with multiple functionalities of flexibility and sensing modalities to force, temperature and so on, have been termed sensitive skin, smart skin, or electronic skin (E-Skin) [6]. The difference between H-skin and E-Skin is that the former has the functionality of photovoltaics, while the latter does not. Since H-skin also has piezoelectric properties, it can use solar power for self-sensing by utilizing the generated Piezo effect. The novel artificial skin is effective in futuristic applications for humanoid and the outer layer of robot skin. Its fabrication and properties are detailed below.

In the process of development of an elastic solar cell, a new method was used for vulcanizing natural rubber by electrolytic polymerization, together with a configuration of magnetic clusters of metal particles incorporated into the rubber by the application of a magnetic field [7]. This material is named magnetic compound fluid (MCF) rubber, which was reported in a previous study. As for MCF, many engineering applications in dampers and polishing machines have been proposed with MCF as other applications [8], since it is an effective magnetic-responsive intelligent fluid, unlike magnetic fluid (MF) and magneto-rheological fluid (MRF), which were devised in 2001. MCF rubber is not only elastic, flexible, and extensible, but it is also sensitized to piezoelectricity and piezoresistivity [9], where the piezoelectricity is due to the built-in voltage generated in the material due to change in the distance between the ions in the material under compression. The piezoresistivity is attributed to the resistance of the material caused by the change in electric current due to the application of voltage under compression. This mechanism results from the conductivity of the material.

The production methods and power-generation principles of ordinary solid-state solar cells, like photoexcitation based on p- and n-type semiconductors, photosensitized dye molecules and so on, are relevant and effective for the preparation of an elastic and extensible solar cell with rubber. It is not intrinsic semiconductors, but doped semiconductors, which are used for preparing the solar cell. By the doping process, the p- and n-type semiconductors are ionized. The former is an acceptor, A, which is charged negatively by accepting an electron (denoted by A^−^); the latter is donor, D, which is charged positively by giving an electron (denoted by D^+^). As the MCF rubber has A^−^ and D^+^ ions by electrolytic polymerization, it attains photovoltaic properties, as shown by the electrolytic polymerization reaction in the following section. The photosensitized dye molecules yield two types of MCF rubber solar cells: The dye displays photoexcitation or photoactivity when it is compounded with MCF rubber, which on vulcanization results in arid MCF rubber solar cell (dry type); when the dye seeps into the electrolytically polymerized MCF rubber, the photovoltaics are generated by a redox reaction, then the MCF rubber solar cell is wet (wet type). The former is closer to the concept of a perovskite-type solar cell [10] and is dealt with in this report, while the latter refers to a novel dye-sensitized solar cell [11,12] or Gratzel-type solar cell [13] and will be discussed later. These principles and concepts are useful for the production of the MCF rubber solar cell suggested in this study.

In the present study, the method of production of soft solar MCF rubber having photovoltaic and piezoelectric properties and using a chemical-physical model, was investigated. In the beginning, the principle of generation of photovoltaics in MCF rubber solar cell is described. Next, the photovoltage and current due to photoexcitation, based on p- and n-type semiconductors resulting from the electrolytic polymerization of MCF rubber, by doping or due to the dye, were measured. The effect of compression on the properties of the dry-type MCF rubber solar cell was studied. The photovoltage, photocurrent density, and piezoelectric sensing were measured to ascertain the photovoltaics due to irradiation by visible light under compression.

## 2. Principle of a Dry-Type MCF Rubber Solar Cell

**Figure 1:**
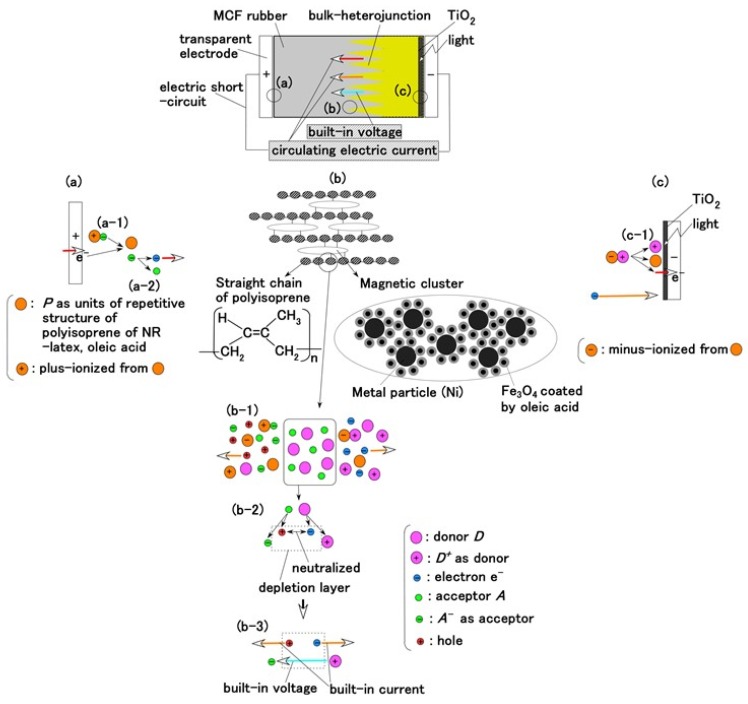
Model of electrolytically polymerized MCF solar cell rubber vulcanized in a magnetic field: (**a**) response at the area facing anode side with transit from (**a-1**) to (**a-2**) which is generated by irradiation; (**b**) response at the area between anode and cathode sides which is shifted from (**b-1**) to (**b-3**) as built-in voltage and current; (**c**) response at the area facing cathode side with transit (**c-1**) which is generated by irradiation.

A compound MF, consisting of other metal particles such as Ni, of size of the order of 1 μm is an MCF, one of the intelligent fluids responsive to a magnetic field. The MCF in the present study is a colloidal fluid composed of 10 nm Fe_3_O_4_ particles coated with oleic acid having 5 nm thickness for uniform dispersion because MF consists of Fe_3_O_4_ particles coated with oleic acid. Each Fe_3_O_4_ particle coated by oleic acid is dispersed in a solvent of water. The configuration of the oleic acid is the same as the one of ordinary magnetic fluid (MF). Due to the hydrophilic group of the oleic acid, whose molecule is easily wetted by water, it is effective for water diffusion. Therefore, MCF rubber liquid has water-based solvent so that the oleic acid is stable in the solvent. We can consider three parts of the MCF rubber solar cell as shown in Figure 1a–c. 

The configuration of cross-linked polyisoprene on the electrolytically polymerized MCF rubber, compounded with natural rubber (NR-latex), as shown in Figure 1b [7], was elucidated in a previous study [14]. NR-latex is ordinary rubber with greater elasticity and compressibility, so that the H-Skin conforms to the purpose of the present study [7]. It must be understood that the reactions shown in Figure 1a–c occur at the microscopic level among the particles of Fe_3_O_4_ and Ni, and the molecules of polyisoprene, which are dispersed in a water solvent. When a magnetic field is applied, the Fe_3_O_4_ particles play a bonding role among the metal particles of Ni, causing numerous Ni and Fe_3_O_4_ particles to aggregate into a magnetic cluster of needle-like shapes as shown in Figure 1b [7], which can be ascertained by using the technique of extraction of magnetic clusters from magnetic responsive intelligent fluids, devised in 2003 [15]. The existence of magnetic clusters in the MCF rubber facilitates the development of a solar cell with a bulk-heterojunction of alternating junctions of multi-layered donor and acceptor materials for higher solar cell efficiency, unlike the case of ordinary organic thin-film solar cells in which it is difficult to obtain the configuration. 

When the NR-latex compounded with MCF is electrolytically polymerized in a magnetic field applied in the same direction as the electric field lines, the polyisoprene NR-latex molecules align along the magnetic field lines and crosslink each other. This is due to the induced electrochemical reaction occurring around the anode, given by Equation (1), analogous to the chemical analysis by X-ray photoelectron spectroscopy (XPS) and so on, and the electrochemical analysis by oxidation-reduction potential (ORP) and pH [14]. On the other hand, the oleic acid and polyisoprene are crosslinked, as shown in Figure 1b, by the electrochemical reaction given by Equation (2), which occurs around the cathode.

Three types of dry-type MCF rubber solar cell were investigated: (A) MCF rubber without photosensitized dye molecules, TiO_2_ as an electron transport material or KI + I_2_ (which is mixed by potassium iodide (KI) and iodine (I_2_)) as an electrolyte, that are commonly used in ordinary dye-sensitized solar cells [11,12]; (B) MCF rubber with just TiO_2_; and (C) MCF rubber with dye and KI + I_2_ as well as TiO_2_. These are based on the following concepts: (A) polyisoprene exhibits photovoltaic effect because C=C bonds display photoexcitation; (B) TiO_2_ has the role of an n-type semiconductor as a donor, and that the photovoltaic effect of (B) is the one with which the one of (A) is added; (C) the dye undergoes photoexcitation or photoactivity, K^+^ of KI has the role of an n-type semiconductor as a donor, and iodide/triiodide (I^−^/I_3_^−^) of KI + I_2_ plays the role of a p-type semiconductor as an acceptor, and that the photovoltaic effect of (C) is the one with which the ones of (A) and (B) are added. In each of the types (B) and (C), the dye, TiO_2_, and KI + I_2_ are compounded in the MCF rubber and electrolytically polymerized to be dried and vulcanized in slightly wetter conditions. In all the types (A), (B), and (C), the solar cell has a cathode of transparent glass coated with a thin TiO_2_ layer on which the light is incident, similar to an ordinary dye-sensitized solar cell. 

Owing to the incident light at the cathode, a reaction is generated as shown in Equations (3a) and (4), respectively. The former is shown in (a-1) at the anode and the latter in (c-1) of Figure 1 at the cathode: at (c-1), the electron is given to the cathode and at (a-1), from the anode. As shown in (a-2) of Figure 1, electrons move away from A^−^ and pass through the MCF rubber, corresponding to Equation (3b). The terms in Equations (3) and (4) correspond to those in Equations (1) and (2), respectively: D^+^Py^−^ in Equation (4) corresponds to the right-hand side (RHS) of Equation (2), P, to the first and the fourth terms on the left-hand side (LHS) of Equation (2), and D^+^, to the second term of the LHS of Equation (2); Py^+^Ay^−^ in Equation (3) corresponds to the first term of the RHS in Equation (1), P, to the first term of the LHS in Equation (1), and A^−^, to the second term of the LHS in Equation (1).

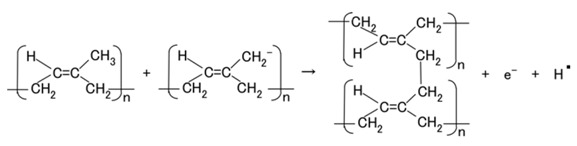
(1)

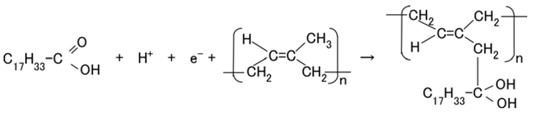
(2)

(3a) $${\left[P^{y+}A_{y}^{-}\right]}_{x}+{\mathrm{xye}}^{-}\to P_{x}+{\mathrm{xy}\;A}^{-}$$

(3b) $${\mathrm{xyA}}^{-}\to A+{\mathrm{xye}}^{-}\;\mathrm{at}\;\mathrm{anode}\;\mathrm{of}\;\mathrm{solar}\;\mathrm{cell}\;\mathrm{side}$$

(4) $${\left[D^{+}P^{y-}\right]}_{x}\to P_{x}+{\mathrm{xy}\;D}^{+}+{\mathrm{xye}}^{-}\;\mathrm{at}\;\mathrm{cathode}\;\mathrm{of}\;\mathrm{solar}\;\mathrm{cell}\;\mathrm{side}$$

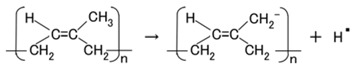
(5)

In type (A), A^−^ is the negatively ionized polyisoprene P, and polyisoprene is an anion with a radical reaction in the normal state of NR-latex, as shown by Equation (5). In types (B) and (C), A^−^ corresponds to P^−^ and iodide/triiodide (I^−^/I_3_^−^); D^+^ to the ionized TiO_2_ and K^+^.

It can be understood from Equations (1), (2), and (5) that the water molecule is a significant component. NR-latex is a water-soluble rubber and an aqueous medium is used for the solar cell with a transparent glass electrode coated with TiO_2_; hence, the effect of water molecule on a photovoltaics is generated in the MCF rubber solar cell, called the Honda–Fujishima effect [16] designated by Equation (6), with D and A under the catalyst of anionic polyisoprene (as the first term of the RHS in Equation (5)) and radical oleic acid (as Equation (7)). This reaction has been discussed in the field of macromolecular complexes, and because MCF contains metal particles (Ni in the present study), it is similar to a polymer-metal complex. Therefore, the physical interpretation of macromolecular complexes can be assumed according to which the photovoltaic effect of polyisoprene is due to the sequential potential field along C=C bonds, which can be found in other polymer-metal complexes [17]: the path and orientation of the electron-transfer reaction gives rise to the photoexcitation and is defined by a sequential potential field. The electron-transfer reaction is also caused by other sequential potential fields between D and A, as shown by Equations (3), (4), and (6)—the electron can move easily from higher to lower potential.

(6) $$\begin{array}{c} D+A↔\left(\mathrm{DA}\right)↔{\left(\mathrm{DA}\right)}^{*}↔{\left(D^{+}A^{-}\right)}^{*}↔\left(D^{+}A^{-}\right)↔D^{+}+A^{-}\\ 4D^{+}+2H_{2}O\to 4D+O_{2}+4H^{+}\\ 2A^{-}+2H_{2}O\to 2A+H_{2}+2{\mathrm{OH}}^{-} \end{array}$$

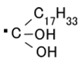
(7)

This can be explained by using the electron transfer theory in the field of macromolecular complexes. After the dispersion of the particles of Fe_3_O_4_ and Ni, and molecules of polyisoprene, the interaction among these is varied. When the distance between them is large, the interaction is comparatively weak and the photovoltaic reaction of Equations (3) and (4) can be considered as an outer-sphere electron transfer reaction (OSETR), which means the structural coordination of molecules is not deformed and only the electrons are transferred by the tunneling effect. In contrast, there can be an inter-sphere electron transfer reaction (ISETR) in the case of a comparatively strong interaction among them due to the following reasons: There is an inherent reaction, as shown by Equation (8) indwelling in Equation (1), where Br is given by Equation (9) and RH by the LHS in Equation (5) [14]. From Equation (8), the anionic isoprene, R, can be considered a bridging ligand and Equation (1) can be considered to be an ISETR, generated on the basis of the interaction of the reactants and mediation of the bridging ligand. On the other hand, Equation (2) is divided into Equations (10) and (11), where anionic oleic acid can be considered a bridging ligand; hence, Equation (2) can be an estimated ISETR. Whether OSETR or ISETR is generated depends on the probability of the distance among the particles of Fe_3_O_4_ and Ni, and molecules of polyisoprene—OSETR takes place in the case of long distances and ISETR for smaller ones. As numerous particles and molecules are dispersed in a solvent, many such OSETRs or ISETRs must be taken into account.

(8) $$∙\;\mathrm{Br}+\mathrm{RH}\to \mathrm{Br}\dot{R}H\to \mathrm{RBr}+H^{●}$$

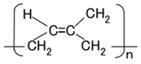
(9)

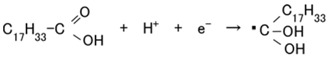
(10)

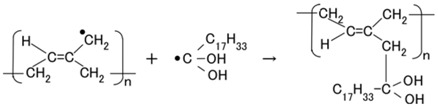
(11)

In type (C), the dye is excited by the incident light and the electron is emitted by the cathode. The ionized dye is reduced by an electron as shown in Equation (12), with sequential reaction as shown in Equation (13). As a result, a photocurrent is generated.

(12) $$2\;{\mathrm{Dye}}^{+}+2\;I_{3}^{-}\to 2\;\mathrm{Dye}+3I_{2}\;\mathrm{at}\;\mathrm{cathode}\;\mathrm{of}\;\mathrm{solar}\;\mathrm{cell}\;\mathrm{side}$$

(13) $$3I_{2}+2\;e^{-}\to 2\;I_{3}^{-}\;\mathrm{at}\;\mathrm{anode}\;\mathrm{of}\;\mathrm{solar}\;\mathrm{cell}\;\mathrm{side}$$

Meanwhile, before illumination, when a microscopic part of the adjacent particles or molecules, as shown in (b-2) of Figure 1, is viewed, D and A appear to be ionized. A depletion layer area is developed due to the neutralization of the electrons and holes that originated from D and A, respectively; a voltage is generated between D^+^ and A^−^ as built-in voltage and an electric current by the remnants of electrons and holes as built-in current, which is the same phenomenon as that occurring in the case of common piezoelectricity, as shown in (b-3) of Figure 1. The numerous particles of Ni, Fe_3_O_4_, and NR-latex, and the molecules of water, acceptor A, and donor D are dispersed in a jumbled state as shown in (b-1) of Figure 1. Finally, the photocurrents, built-in voltage, and current generated by the above-mentioned reactions were measured indicating the possibility of acquiring both photovoltaics and piezo-sensing. This is shown in the following section on experimental results.

## 3. Materials and Experimental Methods

**Figure 2:**
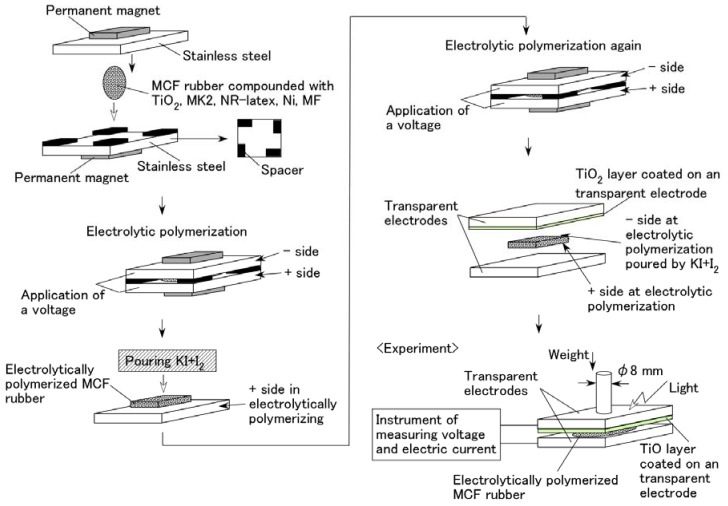
Schematics of the MCF rubber production procedure to create solar cell.

Figure 2 illustrates the schematics of the production procedure for an MCF rubber solar cell. A 0.2 g n-type semiconductor TiO_2_ and 0.02 g dye sensitizer MK2 (metal-free solar cell dye, C_58_H_70_N_2_O_2_S_4_, Sigma-Aldrich Japan Co. LLC., Shimomeguro, Japan) were compounded with MCF rubber, which consisted of 0.6 g carbonyl Ni powder, with particles on the order of micrometers and bumps on the surface (No. 123, Yamaishi Co., Ltd., Noda, Japan), 0.45 g water-based MF with 40 wt % Fe_3_O_4_ (W-40, Ichinen-Chemicals Co., Ltd., Shibaura, Japan), and 1.8 g NR-latex (Rejitex Co., Ltd., Atsugi, Japan). These components were mixed as stated for each type of (A), (B), and (C) viz. A constant electric field was applied at 6 V, and an electric current of 2.7 A was passed between stainless-steel plates with a 1 mm gap for 10 min under atmospheric conditions and application of a 188 mT magnetic field across the liquid. While using MK2, two cases of electrolytic polymerization were considered viz. After electrolytic polymerization without KI + I_2_, 0.17 g solution KI + I_2_, compounded with 3.3 g I_2_ in a solution of 40 g potassium iodide KI, and 60 g water, was poured on one side of the electrolytically polymerized MCF rubber as an electrolyte, and the rubber was electrolytically polymerized again (twice polymerization); MCF rubber was once electrolytically polymerized with KI + I_2_ (once polymerization). 

The final electrolytically polymerized MCF rubber was around 20 mm × 23 mm × 1 mm in size. It was sandwiched between a transparent glass electrode and a TiO_2_-coated one. Visible light (238 Lux) and ultraviolet light (227 Lux) were scattered on the transparent electrode coated with TiO_2_. As shown in the bottom left of Figure 2, a weight was applied on one side of the electrode under the incident light. The rod with a diameter of φ8 mm installed the underneath of the weight was attached to the electrode. Voltage and electric current between the electrodes were measured using a digital multi-meter (PC710, Sanwa Co. Ltd., Fukuoka, Japan). The MCF rubber produced using the present procedure of electrolytic polymerization was of dry type because of dehydration to some extent owing to the heat of the electrolytic polymerization, in contrast to an ordinary dye-sensitized solar cell [10,11], where the dye sensitizer and electrolyte are involved in the MCF rubber. Therefore, the present MCF rubber solar cell was different from that in a fluidic state due to the dye and electrolyte being poured on it.

## 4. Results and Discussion

### 4.1. Without Compressing

**Figure 3:**
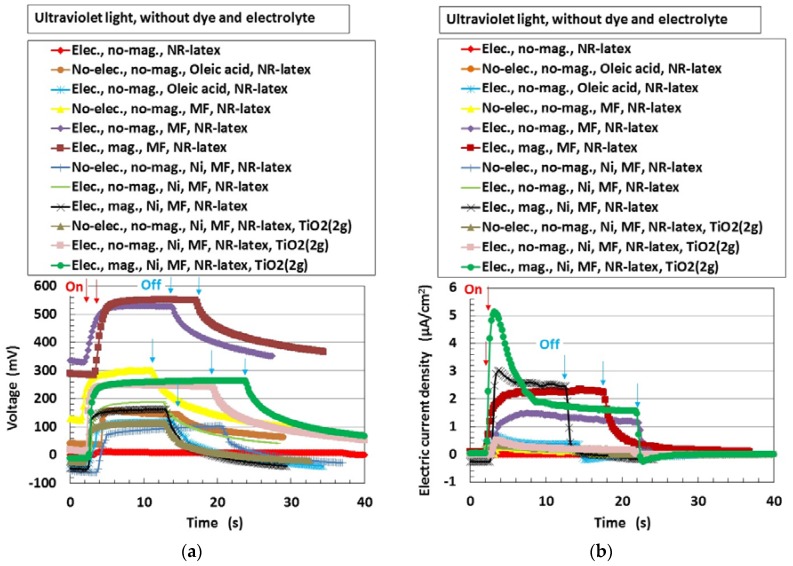
Change in voltage and electric current density by turning ultraviolet light on and off: (**a**) voltage; (**b**) electric current density. “elec.”, electrolytic polymerization; “mag.”, with magnetic field at electrolytic polymerization; “no-“, without one. Each color indicates a component of the MCF rubber solar cell made of types (A) and (B), without MK2 and KI + I_2_.

First, the photovoltaics, without compression, were examined in order to understand the physical mechanism of the MCF rubber solar cell. Figure 3 shows the changes in voltage and electric current density when ultraviolet light was turned on and off, designated by colored arrows in the figure. The data are for types (A) and (B), without MK2 and KI + I_2_. They exhibited photoelectricity (photovoltage and photocurrent), including built-in electricity (built-in voltage and current). The former was due to a change of start-up voltage or electric current due to incident light and the latter was the initial voltage or electric current. The built-in voltage suggests the occurrence of the piezoelectric effect. In addition, due to the reason described in (b-3) of Figure 1, built-in current also occurred when these were removed initially in some cases of the data of Figure 3. The cause was the integrated data at microscopic part of adjacent particles or molecules, of which the state corresponded to dispersion as shown in (b-1) of Figure 1. 

The magnitude of photoelectricity, excluding the built-in electricity, due to irradiation by visible and ultraviolet light is shown in Table 1.

The photoelectricity was very small in the case of electrolytically polymerized NR-latex without the application of magnetic field. Photoexcitation owing to C=C bonds in polyisoprene was very minimal. In contrast, it is clear that the mixture of NR-latex and oleic acid was photovoltaic, whether it was electrolytically polymerized or not, by looking at the case of “Oleic acid, NR-latex”. This was due to the ionized polyisoprene, which (as shown by the second term on the LHS of Equation (1)) played the role of an acceptor-like p-type semiconductor (corresponding to A^−^ in (a-1) of Figure 1), and due to the hydrogen ion decomposed from water molecules in NR-latex and MF (see second term in LHS of Equation (2)), which played the role of donor-like n-type semiconductor (corresponding to D^+^ in (c-1) of Figure 1). Therefore, the NR-latex and oleic acid in MCF rubber produced a photovoltaic effect. In addition, since MF included oleic acid, the mixture of NR-latex and MF was photovoltaic, by looking at the case of “MF, NR-latex”. The results show that oleic acid had a role of catalyst as a bridging ligand on ISETR in the field of macromolecular complex [18], which was the effect of oleic acid on the MCF rubber solar cell.

On the other hand, like the configuration of p- and n-type semiconductors, and the formation technique of organic photoelectric conversion elements in an ordinary solid-state solar cell such as an organic polymer film solar cell, many magnetic clusters indicated by (b) in Figure 1 form the bulk hetero-junction structure. Although the MCF rubber solar cell is a mixture of p- and n-type semiconductors, etc., unlike the layered structure of the components in a solid-state solar cell, the bulk hetero-junction structure exists as shown in (b) in Figure 1. Therefore, focusing on the cases of “MF, NR-latex” and “Ni, MF, NR-latex”, photocurrent density in “mag.” decreased more than that in “no-mag”.

The comparison between the cases of “MF, NR-latex” and “Ni, MF, NR-latex” denotes the effect of metal particle on photoelectricity. There is a possibility that Ni reacts with surface hydroxyl group of oleic acid of MF as in Equation (14). The results that carbonyl group of Ni has a role of catalyst well-known at the field of macromolecular complex [19] provides the effect of Ni on MCF solar cell rubber.

(14) $$\mathrm{Ni}+2\;\mathrm{OH}\to {\mathrm{Ni}}^{2+}+2O^{-}+H_{2}$$

In general, TiO_2_ is most often used in an ordinary dye-sensitized solar cell as an electron transport material [10,11]. The interfacial electron-transfer process of TiO_2_ in colloidal semiconductor systems has been reported [20]. Therefore, it is understandable that TiO_2_ affects the photovoltaics, by looking at the case of “MF, NR-latex, TiO_2_ (2 g)” in Figure 3b.

In the case without electrolytic polymerization, indicated by “No-elec.” in Figure 3, Equations (3a) and (4) are replaced by Equations (15) and (16), respectively.

(15) $${\left[P^{y+}A_{y}^{-}\right]}_{x}\to P_{x}+\mathrm{xyA}\;\mathrm{at}\;\mathrm{anode}\;\mathrm{of}\;\mathrm{solar}\;\mathrm{cell}\;\mathrm{side}$$

(16) $${\left[D^{+}P^{y-}\right]}_{x}\to P_{x}+\mathrm{xyD}\;\mathrm{at}\;\mathrm{cathode}\;\mathrm{of}\;\mathrm{solar}\;\mathrm{cell}\;\mathrm{side}$$

In Figure 3, the curves of voltage and electric current density tended to slope downwards gradually when the light is turned off, compared to their trend when it was turned on. This can be explained by using the theoretical formation of the rate of electron-transfer reaction obtained from electron-transfer theory [21]. On the other hand, the tendency of the curves to slope downwards suddenly when the light was “off” and then recover slightly to become constant, in some cases, can be explained by using the long-range electron-transfer theory [22]—it is due to the changes in pH of the MCF rubber solar cell due to change in [H^+^] and [OH^−^] under the reactions of Equation (1), (2), (5), (6), and (14). When the potential at the particles of Fe_3_O_4_ or Ni or molecules of polyisoprene becomes equivalent to that at an adjacent one from the state where their potentials are different, electron transfer is reversed because the electron can move to the orientation of smaller potential. The electron transfer is explained under the state of OSETR. 

In addition, the photovoltaic property of the MCF rubber solar cell resulted in a redox reaction as shown by Equations (1)–(4) and (6). The possibility of the above processes can be confirmed in an ordinary dye-sensitized solar cell, such as the Gratzel-type solar cell.

It can be seen from Table 1 that there was an increase in the photoelectricity when oleic acid, MF, or TiO_2_ were added to NR-latex, during the process of electrolytic polymerization in the magnetic field. It was greater for ultraviolet light than for visible light because TiO_2_-coated transparent glass reacts with the former: TiO_2_ is a typical material reactive to ultraviolet light.

**Figure 4:**
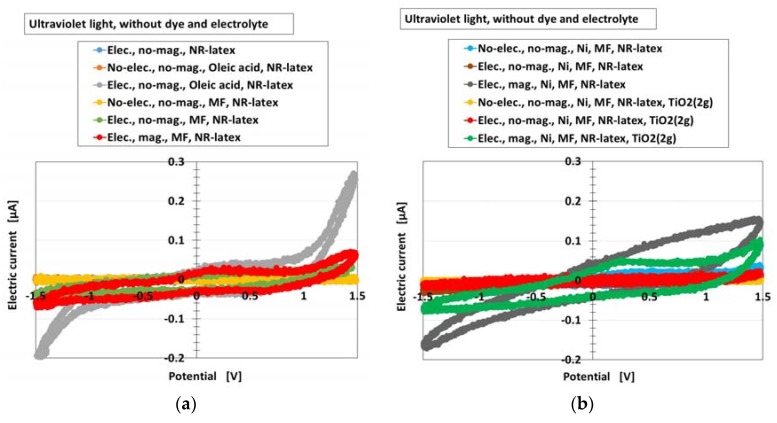
Current-potential curves of the MCF rubber solar cell of types (A) and (B) (these types denote in Section 2) without MK2 and KI + I_2_ by turning ultraviolet light on, for each of the components of Figure 3: (**a**) for cases of NR-latex, NR-latex with oleic acid, NR-latex with MF; (**b**) for cases of NR-latex with Ni and MF, NR-latex with Ni, MF and TiO_2_. “elec.”, electrolytic polymerization; “mag.”, with magnetic field at electrolytic polymerization; “no-”, without one. Each color indicates a component of types of (A) and (B) of the MCF rubber solar cell without MK2 and KI + I_2_.

Furthermore, the photovoltaic characteristics of the MCF rubber solar cell were investigated by an analysis of the current-potential curves. Figure 4 shows these curves for the types (A) and (B) of the cell without MK2 and KI + I_2_ under illumination by ultraviolet light, for all the components of Figure 3. They were measured by a potentiostat (HA-151B, Hokuto Denko Co. Ltd., Tokyo, Japan) at 50-mHz scan rates in the potential domain of −1.5–1.5 V. It can be seen that the electric current changed rapidly at around the largest positive or negative potential for the component “Elec., no-mag., Oleic acid, NR-latex”. This indicates a photodiode property and testified that MCF rubber had this property, derived from the combination of n- and p-type semiconductors corresponding to D and A, respectively, which was previously denoted in Section 2. The photodiode effect can be seen in organic optoelectronic devices [23] and luminescent solar concentrators made of polysiloxane rubber [4]. However, there are few studies on photovoltaics of rubber-state photodiode. Therefore, the results obtained in the present study are of great value.

On the other hand, as seen from all the results of the different components, the swelling of the curves was noticeable. It indicates that there was more photoinduced oxidation and reduction, which was previously denoted in Section 2. As a similar tendency can be seen with photocatalyst TiO_2_ [24], the reaction previously denoted in Section 2 is validated.

**Figure 5:**
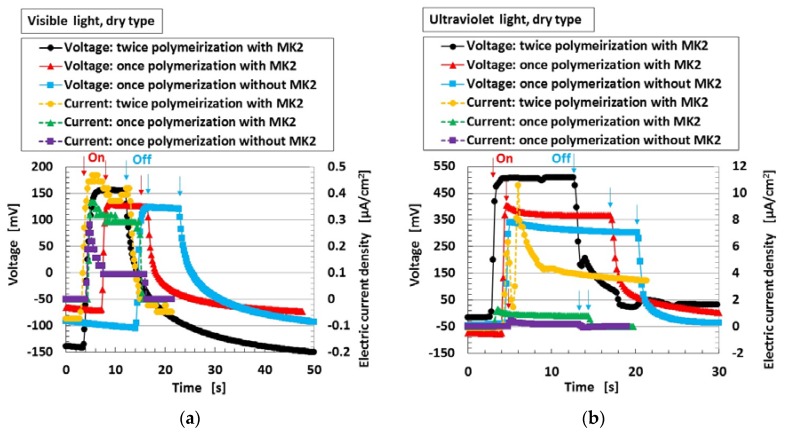
Change in voltage and electric current density: (**a**) by turning visible light on and off; (**b**) by turning ultraviolet light on and off. Each color indicates a component of the MCF rubber solar cell of type (C).

Next, Figure 5 shows the changes in voltage and electric current density when visible and ultraviolet light were turned on and off, designated by colored arrows in the figure for type (C) with MK2 and KI + I_2_. By doping with the dye sensitizer MK2, the photoelectricity was generated naturally. The operation of TiO_2_, dye, and iodide/triodide (I^−^/I_3_^−^) was the same as in an ordinary solid-state dye-sensitized solar cell [10,11]. There were no major differences in the photovoltaic effect in cases of both “once polymerization” and “twice polymerization”.

Finally, we compare photovoltaic characteristics between commercial solar cell and the MCF rubber solar cell. Ordinary solar cells are generally categorized three types: silicon, compound, and organic. The commercial silicon solar cell has mA-ordered photocurrent, however, organic thin film solar cell, which involves well-known perovskite-type solar cell [10] and dye-sensitized solar cell [11,12], such as Gratzel-type solar cell [13], has scores of μA ordered photocurrent. Dry-type MCF rubber solar cell has μA-ordered photocurrent, in contrast, wet-type MCF rubber solar cell that was shown in the consecutive second report had scores of μA ordered photocurrent, which was the same ordered photocurrent of the ordinary organic thin film solar cell. Regarding photovoltage, the three types of the categorized solar cells, as well as dry and wet types of MCF rubber solar cells, have hundreds of voltage. On the other hand, the response time to illumination of ordinary commercial silicon solar cell is faster than the one of ordinary organic thin film solar cell. The response time of dry and wet types MCF rubber solar cell is the same as the one of ordinary organic thin film solar cell, which is dealt with in the consecutive second report [25].

### 4.2. With Compressing

**Figure 6:**
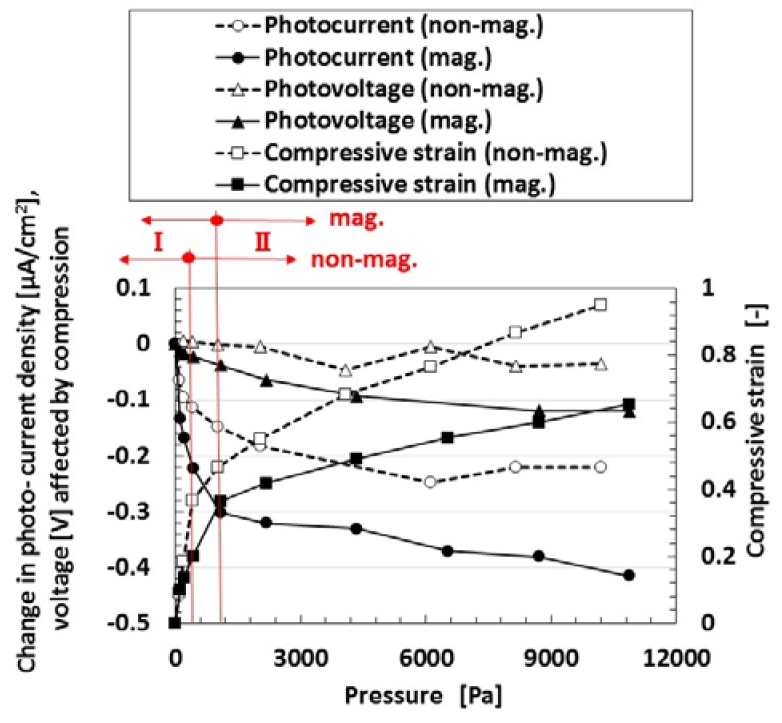
Change in photovoltage and photocurrent by pressure: “mag.”, with magnetic field at electrolytic polymerization; “no-”, without one. Each color indicates a component of the MCF rubber solar cell of type (C).

**Figure A1:**
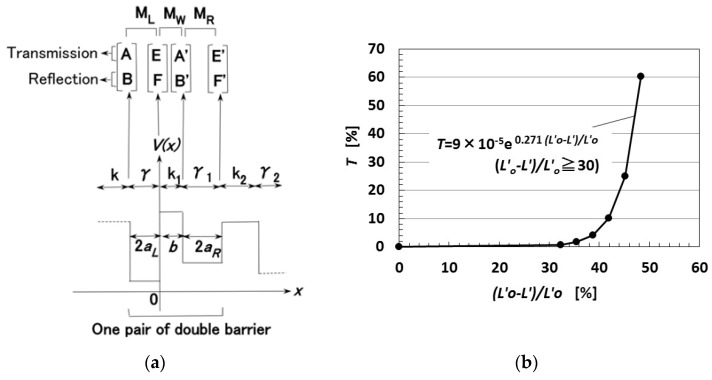
Theoretical results of the change in the translated ratio of electric current in relation to the compression ratio of the MCF rubber [25,29]: (**a**) Model of multi potential barrier of the rubber; (**b**) transmitted probability by compressing.

Finally, we investigate the effect of compression on the photovoltage and photocurrent density due to irradiation by visible light in order to study the photovoltaics involving the piezoelectric effect. Figure 6 shows the photocurrent density and photovoltage extracted from the measured electric current density and voltage in the case of the MCF rubber solar cell of type (C), made by “twice polymerization” under compression. The negatively changing photocurrent density and voltage was due to the change in the initial state, which is caused by the following physical mechanism. Because of the mixture of p- and n-type semiconductors and others, we can understand the position of A^−^ and D^+^ by the dispersion of particles and molecules as shown in (b-1) of Figure 1. The total change in the position of A^−^ and D^+^ occurs in reverse along the electric field lines due to the pressure. From the electron transfer theory in the field of macromolecular complexes, in the case of OSETR with weak interaction among particles of Fe_3_O_4_ and Ni and molecules of polyisoprene, the resonance tunneling theory [26] or superexchange model [27] has been proposed as a well-known theoretical model. The latter is relevant to electron transfer between energy band gap of lowest unoccupied molecular orbitals (LUMO) and highest occupied molecular orbitals (HOMO) and ordinarily utilized to explain photoexcitation in solar cells. However, the tunneling theory is essential to explain the same, numerically. The material gap between p- and n-type semiconductors or A^−^ and D^+^ is an insulator with non-adiabatic process—it is through which an electron can jump to shift between different potential energy regions. The transfer of electric current in an MCF rubber made of silicon oil rubber solidified by drying without electrolytic polymerization has been investigated numerically by using the tunneling theory [28,29]. The same theoretical formula can be applied to the present MCF rubber solar cell. As shown in Figure A1 in Appendix A, transfer of electric current is easier under compression because of the decreasing size of the gap. It explains the electric conductivity in a composite conductor composited in an insulator, or the piezo effect in the state before contact of each conductor by compression. The same shall apply for the piezo effect on the photoelectricity, as shown in Figure 6.

The relation between the pressure and the compressive strain is also shown in the figure. There were two linear compressive regions with compressive elasticity, designated by I and II, and the latter had a slight nonlinearity because of the application of large compression, although, in general, there did not usually exist a plastic region in compression. The rigidity in “mag.” was larger than that in “non-mag.” because of the existence of Fe_3_O_4_ and Ni particles as fillers. The photovoltaic effect was increased by compression from viewing the changes in the absolute values. The changes of the photovoltaic effect become larger with increasing gradient of the compressive strain to pressure and compressive Young’s modulus. The magnitude of the pressure used in the present experiment is important because the maximum pressure corresponds to the used force with 500 g weight, and it is a small pressure range. Regarding the effect of larger pressure on photovoltaic effect, it is dealt with in a consecutive report.

In the case of no magnetic field applied under the electrolytic polymerization, no bulk hetero-junction structure was formed. Therefore, it was confirmed that the photovoltaic effect was increased due to the existence of the bulk hetero-junction structure.

## 5. Conclusions

The feasibility of designing a dry-type soft rubber solar cell by compounding MCF and NR-latex under electrolytic polymerization was investigated experimentally using a chemical-physical model with and without an electrolyte and a dye sensitizer. Photoexcitation was caused by the ionized polyisoprene in the role of an acceptor-like p-type semiconductor, and the hydrogen ion in the role of a donor-like n-type semiconductor, to create electrolytic polymerization in MCF rubber. It was just the mixing of the p- and n-type semiconductors, dye, and others, in an MCF rubber solar cell that caused the photovoltaics to manifest. By the application of a magnetic field under electrolytic polymerization, many magnetic clusters formed a bulk hetero-junction structure, enhancing the photovoltaics, whether the solar cell was compressed or not. In addition, the MCF rubber solar cell displayed piezoelectricity because of the built-in voltage and electric current from the ionized acceptors and donors. The photovoltaic effect was increased by compression. However, the result occurred within a small pressure range.

The electric current density obtained in the present experiment was relatively small. The causes were several experimental conditions—the kind of sensitized dye; the difference from the wet-type MCF rubber solar cell; the work function of the electrode material. These are dealt with in another consecutive report.

It is effective to utilize MCF rubber for enabling compression of a solar cell, as it is able to deploy the features of both piezoelectricity and photovoltaics in it. Therefore, it is shown that the H-Skin displays the hybrid functions of piezo and photovoltaic effects.

## Figures and Tables

**Table 1:** Photoelectricity by visible and ultraviolet lights for each components of Figure 3. The MCF rubber solar cell was made of types (A) and (B), without MK2 and KI + I_2_. Each specimen denotes circle (○) and slash (/): the former indicated the use of the corresponding component, while the latter indicated no use.

Ni	MF	Oleic Acid	TiO_2_ (2 g)	NR-Latex	Elec.	Mag.	Visible Light		Ultraviolet Light	
							Voltage [mV]	Current Density [μA/cm^2^]	Voltage [mV]	Current Density [μA/cm^2^]
				○			6.31	0.000	13.0	0.000
		○		○			109	0.100	120	0.017
		○		○	○		103	0.138	157	0.759
	○			○			127	0.080	179	0.320
	○			○	○		109	0.317	200	1.18
	○			○	○	○	136	0.594	268	2.25
○	○			○			78.1	0.125	166	0.438
○	○			○	○		98.8	0.173	198	0.317
○	○			○	○	○	105	0.691	215	3.30
○	○		○	○			78.7	0.133	140	0.333
○	○		○	○	○		122	0.162	236	0.541
○	○		○	○	○	○	150	0.799	276	5.10

## Appendix A

Consider the behavior of electrons transmitted over the potential barrier of rubber as a one-dimensional Schrödinger equation in the *x*-direction, which is the direction of compression of the MCF rubber. The process of the transmitting electric current, through a pair of metal regions of *γ* with a range of 2*a_L_* and of *γ*_1_ with a range of 2*a_R_*, and one rubber region *k* with a range of *b* can be considered as a tunnel barrier problem on double barriers, and the diagram of potential energy *V* is shown in Figure A1a [25,29]. MCF rubber is organized as *n* pairs of two metal and one rubber regions. The wave function, Ψ, is presented by Equation (A1), where *ħ* is the *h*/2π, *h* the Planck’s constant, *m* the mass of electron, *V_o_* the potential energy at regions *γ*, *γ*_1_ or *k*, and *ε* the energy of the electron.

(A1) $$-\frac{\hbar^{2}}{2m}\frac{\partial^{2}\Psi }{\partial x^{2}}+V_{o}(x)\Psi (x)=\varepsilon \Psi (x)$$

From Equation (A1), Ψ is resolved as Equations (A2) and (A3), respectively, for the regions *k* and *γ* where *A*, *B*, *C*, and *D* are arbitrary constants that can be defined from the continuity of differential coefficients on the boundary between *k* and *γ* (*γ*_1_) regions as a well-known ordinary quantum problem, as follows:

(A2) $$\Psi (x)=Ae^{ikx}+Be^{-ikx};$$

(A3) $$\Psi (x)=Ce^{\gamma x}+De^{-\gamma x},$$

where

(A4) $$k=\sqrt{\frac{2m}{\hbar^{2}}\varepsilon },\gamma =\sqrt{\frac{2m}{\hbar^{2}}(V_{o}-\varepsilon )}.$$

On the one pair of double barriers shown in Figure A1a, the arbitrary constants *A* and *B* exist in relation to constants *E*’ and *F*’ as shown in Equation (A5).

(A5) $$\begin{array}{l} \left[\begin{array}{c} A\\ B \end{array}\right]=M_{L}M_{W}M_{R}\left[\begin{array}{c} E^{\prime }\\ F^{\prime } \end{array}\right],\\ {M_{T}=M}_{L}M_{W}M_{R} \end{array}$$

where

(A6) $$\begin{array}{l} M_{L}=\left[\begin{array}{cc} m_{L11}e^{i\theta_{L11}} & m_{L12}e^{i\theta_{L12}}\\ m_{L12}e^{-i\theta_{L12}} & m_{L11}e^{-i\theta_{L11}} \end{array}\right],\\ M_{W}=\left[\begin{array}{cc} e^{-ik_{1}b} & 0\\ 0 & e^{ik_{1}b} \end{array}\right],\\ M_{R}=\left[\begin{array}{cc} m_{R11}e^{i\theta_{R11}} & m_{R21}e^{-i\theta_{R21}}\\ m_{R21}e^{i\theta_{L21}} & m_{R11}e^{-i\theta_{L11}} \end{array}\right] \end{array}$$

(A7) $$\begin{array}{l} m_{L11}=\sqrt{\frac{1}{4}{\left(1+\frac{k_{1}}{k}\right)}^{2}\cosh^{2}(2\gamma a_{L})+\frac{1}{4}{\left(\frac{{kk}_{1}-\gamma^{2}}{k\gamma }\right)}^{2}\sinh^{2}(2\gamma a_{L})},\\ m_{L12}=\sqrt{\frac{1}{4}{\left(\frac{{kk}_{1}+\gamma^{2}}{k\gamma }\right)}^{2}\sinh^{2}(2\gamma a_{L})+\frac{1}{4}{\left(\frac{k_{1}}{k}-1\right)}^{2}\cosh^{2}(2\gamma a_{L})},\\ m_{R11}=\sqrt{\frac{1}{4}{\left(1+\frac{k_{2}}{k_{1}}\right)}^{2}\cosh^{2}(2\gamma_{1}a_{R})+\frac{1}{4}{\left(\frac{k_{1}k_{2}-{\gamma_{1}}^{2}}{k_{1}\gamma_{1}}\right)}^{2}\sinh^{2}(2\gamma_{1}a_{R})},\\ m_{R21}=\sqrt{\frac{1}{4}{\left(\frac{k_{1}k_{2}+{\gamma_{1}}^{2}}{k_{1}\gamma_{1}}\right)}^{2}\sinh^{2}(2\gamma_{1}a_{R})+\frac{1}{4}{\left(\frac{k_{2}}{k_{1}}-1\right)}^{2}\cosh^{2}(2\gamma_{1}a_{R})},\\ \theta_{L_{11}}=-\tan^{-1}\left[\frac{kk_{1}-\gamma^{2}}{(k+k_{1})\gamma }\tanh(2\gamma a_{L})\right]+(k+k_{1})a_{L}\\ \theta_{L_{12}}=-\tan^{-1}\left[\frac{kk_{1}+\gamma^{2}}{(k-k_{1})\gamma }\tanh(2\gamma a_{L})\right]+\pi +(k-k_{1})a_{L}\\ \theta_{R_{11}}=-\tan^{-1}\left[\frac{k_{1}k_{2}-{\gamma_{1}}^{2}}{(k_{1}+k_{2})\gamma_{1}}\tanh(2\gamma_{1}a_{R})\right]-(k_{1}+k_{2})a_{R}\\ \theta_{R_{21}}=\tan^{-1}\left[\frac{k_{1}k_{2}+{\gamma_{1}}^{2}}{(k_{2}-k_{1})\gamma_{1}}\tanh(2\gamma_{1}a_{R})\right]+\pi +(k_{1}-k_{2})a_{R} \end{array}$$

The transmitted probability can be given by Equation (A8).

(A8) $$T={\left|M_{T_{11}}\right|}^{2}$$

One pair of double barriers is expanded to *n* pairs of double barriers as multi-barriers. Therefore, the matrix as shown by Equation (A5) is substituted into the matrix of the neighboring pair of double barriers, and this procedure is repeated to obtain the matrix of *n* pairs of double barriers, where Equation (A9) can be given because the material of each region of *k* and *γ* is the same as that of the other regions. Here, 2*a* is the thickness of metal particles, and *b* is the thickness of rubber between the metal particles. When *eE_o_* is the applied voltage, the voltage *eE* at the regions of *γ* and *k* is given as Equation (A9). Therefore, Equation (A5) is calculated by substituting *V_o_-ε-eE_o_* for *V_o_-ε* of *γ* in Equation (A4).

(A9) $$\begin{array}{l} k=k_{1}=k_{2}=\ldots ,\\ \gamma =\gamma_{1}=\gamma_{2}=\ldots ,\\ a_{L}=a_{R}=a\\ eE={eE}_{o}\{(2n-1)a+2(n-1)b\}/L^{\prime }\;\mathrm{at}\;\mathrm{region}\;\mathrm{of}\;\gamma \;\mathrm{before}\;k\\ eE={eE}_{o}\{2na+2(n-1)b\}/L^{\prime }\;\mathrm{at}\;\mathrm{region}\;\mathrm{of}\;\gamma \;\mathrm{after}\;k \end{array}$$

As the thickness of the MCF rubber is divided into *n* pairs, the thickness of the MCF rubber *L*′ can be given by Equation (A10):

*L*′ = *n*Δ*L*= 2*n*(*a* + *b*) [m]. (A10)

*T* is calculated as shown in Figure A1b, where *L_o_*′ is the initial thickness of the MCF rubber, and *L*′ is the thickness of the MCF rubber on the application of the compression force. As the MCF rubber is compressed, *T* becomes larger nonlinearly.
